# Genetic, environmental and stochastic factors in monozygotic twin discordance with a focus on epigenetic differences

**DOI:** 10.1186/1741-7015-10-93

**Published:** 2012-08-17

**Authors:** Witold Czyz, Julia M Morahan, George C Ebers, Sreeram V Ramagopalan

**Affiliations:** 1 Wellcome Trust Centre for Human Genetics, University of Oxford, Oxford, UK; 2 Nuffield Department of Clinical Neurosciences (Clinical Neurology), University of Oxford, Oxford, UK; 3 Blizard Institute, Queen Mary University of London, Barts and The London School of Medicine and Dentistry, London, UK; 4 London School of Hygiene and Tropical Medicine, London, UK

**Keywords:** Twins, Discordance, Epigenetics, Heritability, Environment

## Abstract

Genetic-epidemiological studies on monozygotic (MZ) twins have been used for decades to tease out the relative contributions of genes and the environment to a trait. Phenotypic discordance in MZ twins has traditionally been ascribed to non-shared environmental factors acting after birth, however recent data indicate that this explanation is far too simple. In this paper, we review other reasons for discordance, including differences in the *in utero *environment, genetic mosaicism, and stochastic factors, focusing particularly on epigenetic discordance. Epigenetic differences are gaining increasing recognition. Although it is clear that in specific cases epigenetic alterations provide a causal factor in disease etiology, the overall significance of epigenetics in twin discordance remains unclear. It is also challenging to determine the causality and relative contributions of environmental, genetic, and stochastic factors to epigenetic variability. Epigenomic profiling studies have recently shed more light on the dynamics of temporal methylation change and methylome heritability, yet have not given a definite answer regarding their relevance to disease, because of limitations in establishing causality. Here, we explore the subject of epigenetics as another component in human phenotypic variability and its links to disease focusing particularly on evidence from MZ twin studies.

## Phenotypic variability and discordance

The extent to which phenotypic traits are heritable has been a subject of scientific interest, at least since Galton's classic twin study design [1]. Twins offer a unique means to study inheritance. Monozygotic (MZ) twins arise from a single zygote, and have always been thought to inherit identical genomic sequences [1], whereas dizygotic (DZ) twins arise from two different zygotes and, just like siblings, share on average 50% identity in their genomic sequence. To assess the relative contribution of genes to a trait, comparisons are made between MZ and DZ twin concordance, with a greater MZ than DZ concordance rate implicating a role for genetics in determining the trait [1]. Phenotypic discordance between MZ twins has traditionally been ascribed to non-shared environmental exposures [2]; however, recent research highlights this as being too simplified an explanation. In this review, we focus on potential sources of MZ discordance.

## *In utero *environment and discordance

There is a range of early environmental factors that need to be considered as potential explanations for MZ twin discordance. In some studies, similar pre-natal and post-natal conditions, for example a shared *in utero *environment or upbringing in one family, have been thought to promote phenotypic concordance, in contrast to non-shared exposures [2,3]. However, the concept of non-shared environment has important practical limitations. It is difficult to unambiguously identify the distinct factors and explain their differential effects on phenotype [3]. For instance, although MZ twins share a single uterus in multifetal pregnancies, they do not necessarily share a common *in utero *environment. Twinning itself is thought to be a rare malformation and a stochastic event, although there exists evidence for familiality [1,4,5]. MZ twins occur in about 3.5 in 1000 pregnancies or 4 in 1000 live births [6,7]. Depending on the time of zygote splitting, MZ twins can be divided into four groups[1]. If the zygote splits within 3 days, the twins are dichorionic and diamniotic (DC DA) (18 to 36% of all MZ births). If splitting occurs after the third but before the seventh day, the twins are monochorionic but diamniotic (60 to 80% of cases) [1,8]. If division occurs between days 7 and 14, the twins are monochorionic and monoamniotic (MC MA), which accounts for 2 to 4% of all MZ twins[8]. Conjoined twins arise when splitting happens after the days 13 or 14 [1,8]. All multifetal pregnancies are more prone to complications (such as fetal malnutrition, growth restriction, and premature birth), with a mortality rate six times higher than for singletons, and a shorter average duration of twin pregnancy (35 weeks) [4,9-17]. Intrauterine growth restriction (IUGR) is a common issue in twin pregnancies, affecting 12 to 47% of all twin pairs [18]. It often leads to discordance in birth weight [18-20], and has been linked with discordance for a range of phenotypes, including height, head circumference, intelligence, language comprehension and expression, fine motor performance, balance, coordination, and visual-motor perception [18,20,21].

The potential causes of IUGR include genetic predisposition, *in utero *crowding, uneven allocation of blastomeres, uneven blood supply, and placental dysfunction (for example, placental abruption, infarcts, stem vessel thrombosis, velamentous insertion of the cord, and single umbilical artery) [18,19,21-23]. Some of these events, such as unequal division of blastomeres or uneven vascularization of the placenta, can be considered as non-shared early exposures, which can be classified, depending on the adopted definition, as environmental or stochastic. IUGR is even more pronounced in MC twins, for whom differences in placental sharing and vascularization lead to occasional unequal blood and nutrient sharing, and, in about 15% of MC diamniotic pregnancies, result in twin-to-twin transfusion syndrome (TTTS) [6,18,21]. MC twins have a higher incidence of congenital heart disorders, and TTTS increases this risk even further [6]. However, even in the absence of TTTS, MC twins are seven times more likely to develop congenital heart disease, and this usually occurs in one twin only [5-7,13,21,24]. The higher risk nature of multiple pregnancies, their proclivity towards complications, and the twin-twin competition for maternal resources increases the probability of a skewed environment affecting the twins *in utero *[25,26].

After birth, any non-shared environmental exposure, such as diet, smoking, toxin exposure and infection, may contribute towards twin discordance [2,3,22,27-31]. Moreover, early phenotypic differences arising in twins could potentially cause shared exposures to have different effects, leading to dissimilarity between the twins.

## *De novo *mutations and genetic mosaicism

It has been assumed that MZ twins are genetically identical, but a wealth of data are accumulating to show that this is not necessarily the case. Mosaicism for *de novo *mutations, retrotranspositions, indels, duplications, and chromosomal rearrangements may play a role in MZ twin discordance [32-44]. The rate for *de novo *base substitutions has been estimated at about 10^-8 ^per base pair per generation, making some genetic differences between adult twins likely [45]. Postzygotic point mutations have been found to be the source of MZ twin discordance in oral-facial-digital syndrome type 1, Joubert syndrome, Van der Woude syndrome, Darier's disease, and neurofibromatosis type 1 while mosaicism for chromosomal abnormalities has been implicated in discordance for conditions such as Turner syndrome, trisomy 21, trisomy 13, skin pigmentation, and sex phenotypes [46-51]. Postzygotic karyotypic mosaicism caused by faulty mitotic division has also been reported in cases of Ulrich-Turner syndrome [22].

Copy number variants (CNVs), which account for a major portion of the genome, are strongly polymorphic and relatively unstable, with mutation rates 100 to 10 000 times higher than those for single base substitutions[52]. Phenotypic discordance in MZ twins may in part be caused by *de novo *mutations of CNVs and CNV mosaicism [32,34,36,53]. Indeed, it has been indicated that *de novo *CNVs may occur at a rate of 10% per twinning event; however, studies have so far failed to link CNV mosaicism to any specific case of phenotypic discordance in MZ twins [40,54-56].

Additionally, unequal exchange of cells during gestation might potentially lead to discordant fetomaternal microchimerism [22].

## Developmental noise and stochasticity

Some variation is inevitable as a result of transcriptional or translational stochasticity, entailed by the random movements of molecules and the complexity of their interactions [57-63]. It should be expected that such noise can lead to markedly different effects under identical environmental conditions [64]. The effect of developmental stochasticity might amass in a drift-like fashion, and thus be more relevant to discordance in complex polygenic traits such as height or weight, which develop over long periods [58]. Stochastic events such as unequal division of the inner mass cells during twinning, or unequal allocation of the developmental markers or precursor cells to different somatic lineages, have been reported as potential sources of discordance in MZ twins [5,37]. Certain cases of twin discordance might potentially be stochastic in origin, however because the causal mechanisms are not thoroughly understood, it is difficult to separate these from environmental effects and gene-environment interactions. Examples include discordance for eye or hair colour and fingerprint profiles, cases of mirror twinning (affecting up to 25% of MZ twins), and major malformations [22].

Certain cases of differential allelic expression (DAE), which result in random monoallelic gene expression, arising as a result of X-inactivation or allelic exclusion in olfactory and pheromone receptor genes, can constitute a mechanism for stochastically driven phenotypic discordance in MZ twins [22,65-70]. Although DAE has also been estimated to affect about 50% of autosomal genes in B-cells, the evidence from MZ twins indicates that the overall degree of DAE is to a certain extent under genetic control. with an estimated 30% of the affected genes showing significant correlation between co-twins [71]. The precise estimates of DAE and its concordance in MZ twins vary. A comprehensive whole genome expression experiment conducted by Baranzini *et al. *indicated that only 1.9% of heterozygous coding loci showed significant evidence for DAE, but out of these, 57% were concordant between the co-twins, still leaving room for stochastic effects [72]; however, their findings were based on a single MZ twin pair [72].

## Epigenetics

Epigenetics was initially a term coined by developmental biologists, and had no immediate link to the issues of epidemiology and heredity [73,74]. Rather, the term described the way in which gene-environment and gene-gene interactions shape a phenotype during development. The concept was developed as an argument for a complex relation between genes and phenotype. Today, epigenetics is used to describe alterations in genomic function, mainly mitotically heritable changes in gene expression that occur through chemical modifications to the structure of chromatin without altering the DNA sequence [2,27,73-76]. There is some limited evidence for transgenerational inheritance of epigenetic changes in mammals, but the scope and mechanisms are under study [3,77].

Cytosine methylation is one of the most well studied epigenetic alterations found in vertebrates [27,73-76]. It occurs at approximately 4 to 6% of the genomic cytosine residues, depending on the cell type [78]. DNA methylation typically occurs in the context of CpG dinucleotides, although this depends on cell type. In fetal fibroblasts, 99.98% of methylated cytosines are located in CpG dinucleotides [78]. By contrast, for embryonic stem cells the proportion reaches about 75%, which highlights the importance of non-CG methylation for gene expression in pluripotent stem cells. Cytosine methylation is mediated by a family of proteins called DNA methyltransferases [73,75,79]. This form of modification is generally associated with transcriptional inactivation. It both physically prevents transcription factors from binding to the DNA, and can also recruit additional factors, such as methyl-CpG binding domain proteins, which can promote repressive histone modifications [27,73,75,76]. However some CpGs remain unmethylated [73]. The precise mechanism by which the differential methylation of CpG dinucleotides occurs is unknown [73]. Recently, hydroxymethylation of cytosines has been discovered, but its biological significance is not yet known.

The second category of epigenetic modifications characteristic of all eukaryotes is the category of covalent alterations to histone proteins [27,73-76]. These affect the N-terminal histone tails and, depending on the position and the type of alteration, can either repress or promote active chromatin conformation [73,76]. The two most important types are acetylation and methylation [76]. The various types of histone modifications have led to the hypothesis of an epigenetic histone code that moderates transcription in response to developmental cues and the environment [76]. Cytosine methylation, histone modifications, and other types of chromatin remodeling all act together in concert, and either reinforce or disable each other through feedback loops [73].

**Figure 1:**
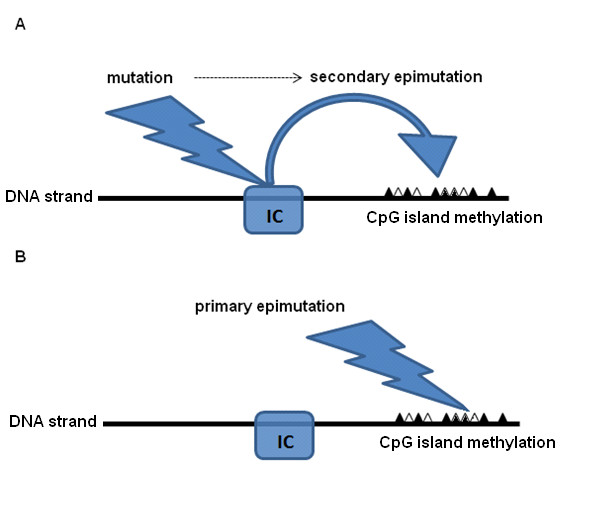
**(A) The mechanism of a secondary epimutation**. A DNA alteration at an imprinting center (IC) indirectly influences and alters the methylation pattern (black and white triangles) at another locus, which could be in *cis *or in *trans*. **(B) **In primary epimutation, the external stimulus (whether environmental or stochastic) directly alters the methylation.

There is substantial evidence in support of epigenetic components in defining human phenotypic variation. Most of this evidence comes from studies of defects in genetic imprinting: an asymmetric, sex-dependent epigenetic moderation of paternal versus maternal gene expression that manifests in monoallelic expression [75]. For imprinted genes, epigenetic modifications are set anew in the germline in each generation, according to sex [75]. Occasional abnormal cytosine methylation can result in epigenetic alterations called epimutations, which just like DNA mutations, can deactivate the gene or cause both copies of the imprinted gene to be transcriptionally active [75]. Such alterations can be divided broadly into three different categories, depending on their origin. Some epimutations have a direct genetic cause, and are secondary to a DNA mutation in *cis *or in *trans*; for instance mutations to imprinting centers. Other epimutations are primary, with no sequence alteration [75,80] (Figure 1). This latter category can be further divided into stochastic epimutations, caused by the inherently error-susceptible molecular machinery, and environmental epimutations, which are caused by environmental factors. This distinction can be arbitrary because the environment can potentially cause stochastic epimutations. The ratio between primary and secondary epimutations is not well defined. There might also exist an intermediate type of 'facilitated epimutations', that is, when the likelihood of a stochastic epimutation at certain locus is increased by genetic determinants[80]. Theoretically, any gene can be targeted by epimutation, giving rise to abnormal conditions; however, imprinted genes represent a more sensitive category on account of their monoallelic expression.

If differences in DNA methylation can be sufficient to cross the boundary between normal and disease phenotype in imprinted, monoallelically expressed genes, then it is also probable that epimutations might affect expression of any pair of genes. Of course, with genes that are expressed in a normal biallelic manner, the effects would presumably be less severe. Consequently, as the complexity and polygenicity of a trait increases, the effect of an epimutation would most probably decrease. However, each potential target for an epimutation would increase the variability of that trait, and because epimutations may arise during development, it follows that organisms can be subject to epigenetic variegation and therefore ought to be heterogeneous for their epigenomes [42,81]. Thus epigenetic mosaicism between twins could be yet another source of phenotypic variation. Indeed, mosaicism in epigenetic alterations has been described in MZ twins, and its relevance to phenotype, particularly disease, is the subject of current studies [82].

The significance of the environment and genes in driving epigenetic changes is a subject of debate, with some authors claiming that epimutations might be stochastic in nature and offer an alternative, non-heritable, and non-environmental explanation for phenotypic variability [2,72,73,83]. The key argument rests on the arbitrary assumption that the random character of *de novo *faults in DNA methylation, whose fidelity is estimated to be at the level of 97 to 99.9% in cell culture, but lower *in vivo*, cannot be ascribed to heritable genetic predispositions or to the environment [2,73]. Thus, the concept of stochastic epimutations as the third source of variation in opposition to genetic and environmental effects has important limitations because it is not evident that the random faults in methylation maintenance are not themselves genetically determined (in a similar way to DAE), or of environmental origin.

Studies using methylome profiling, locally or globally, offer a direct method of evaluating the specific contribution of epigenetics to a phenotype. Some of the main questions raised by these studies' authors concern the nature of this contribution, namely the ratio between hereditary, environmentally triggered, and (potentially independent of the former) stochastic changes. The extent of epigenetic changes and epigenome heritability is disputable. A thorough cross-sectional study of epigenetic profiles in the lymphocytes of 80 MZ twins, aged between 3 and 74 years, revealed significantly greater discordance in the older participants [29]. By estimating total genomic 5-methyl-cytosine content and histone H3 and H4 acetylation, 65% of the twins were found to have almost identical epigenomes, while the remaining 35% were found to be variably discordant. Both the histone acetylation and DNA methylation profiles of twins become progressively discordant with age, different lifestyles, and different medical history. More importantly, the same pattern of epigenetic discordance was seen with buccal epithelial cells, intra-abdominal fat cells, and skeletal muscle cells. Fraga *et al. *(2005) propose that the epigenome is strongly heritable at birth, but epimutations arise and accumulate throughout a lifetime, and their origin arises as a result of a combination of external environmental factors and internal 'epigenetic drift' arising from defects in methylation[29]. Although such maintenance defects have been claimed by others to represent 'endogenous, stochastic mechanisms, independent of environmental perturbations [84], Fraga *et al. *do not exclude the notion they might be environmentally triggered as well [29]. In a similar study, Kaminsky *et al. *(2009) used CpG island microarrays to screen about 6000 loci (as compared with 1800 loci investigated by Fraga *et al.*), in a cohort of 114 MZ and 80 DZ twins, in search of methylation differences [85]. Some discordance was found in white blood cells and replicated in buccal and gut tissue. Estimates based on 20 MZ and 20 DZ pairs indicated that methylation heritability was very low in white blood cells, but rose in buccal tissue (findings based on 19 MZ and 20 DZ pairs), and was significantly greater when dichorionic twins only were considered. The fact that the buccal epithelial tissues of MC MZ twins were significantly more discordant than those of DC twins signals chorionicity as an important environmental factor influencing the epigenome, and this was not taken into account in the study of Fraga *et al. *The sample was not stratified according to age, which precludes inferences regarding early discordance. The findings of Kaminsky *et al. *(2009) may suggest that late twinning can predispose to skewed environmental conditions and to more discordant epigenetic profiles. Kaminsky *et al. *oppose stochasticity to environmentally induced epigenetic differentiation, favoring the former explanation as the more important in phenotypic discordance of MZ twins [85].

The study of Fraga *et al. *(2005) stressed the significance of age in DNA methylation discordance in twins. The youngest twin pair studied by the authors had identical methylation levels [29]. In 2010, Saffery *et al. *sampled four different tissue types from 56 MZ and 35 DZ twins pairs at birth, and analysed them for CpG methylation at four differentially methylated regions (DMRs) associated with the IGF2/H19 locus [86]. Within the MZ pairs, the absolute methylation difference for all DMRs and tissues was generally small and ranged between 3 and 4%; however, the difference varied depending on the tissue type and specific CpG tested. An effect of chorionicity was confirmed. The largely similar epigenetic profiles at birth would support the study of Fraga *et al.*, but conclusions are limited by the investigation of just four genetic regions. Longitudinal studies of CpG methylation in MZ twin cohorts, optimally sampled at birth first, are better means to estimate the levels of epigenetic discordance and make inferences upon its nature. In one of the first longitudinal twin methylation studies, Wong *et al. *attempted to address the issue of epigenetic heritability and stochastic versus environmental epigenetic change [87]. They examined 46 MZ and 45 DZ twin pairs for methylation at three chosen loci relevant to psychology, first at the age of 5 years and then at the age of 10 years. They found variable discordance in all pairs. Not all loci were equally prone to temporal epigenetic change, although alterations were seen for all three genes. By comparing MZ to DZ concordance rates, no significant differences were detected, and the authors concluded that alterations - both shared (indicated by high concordance) and non-shared - were weakly heritable, and thus predominantly attributable to the environment. Interestingly, despite the low heritability, the intraclass correlation coefficients (ICC) of MZ twins remained stable or increased. This might be a consequence of the narrow locus-specific scope of the study [87]. Wong *et al. *did not give any explanation for the phenomenon and concluded that the epigenome is dynamic and subject to changes and environmental influence. It can differ between MZ twins even in early childhood and, depending on the locus, both non-shared and common familial environments can significantly affect its methylation profile. However, Wong *et al. *acknowledge that when ICC and heritability is low, indicating little familial environment and genetic contribution, stochastic epimutations can provide an alternative explanation for the discordance. Further confirmation of those conclusions are required.

The most recent epigenomic study, by investigated methylation levels in a cohort of 230 MZ twin pairs (although 219 pairs appear in the analysis) whose age ranged from 18 to 89 years, both globally and across a panel of nine chosen loci that have been implicated in age-related diseases and epigenetic regulation [88]. The authors adopted a cross-sectional approach, but a subset of 38 twins was re-assayed longitudinally after a 10-year interval. Although a small intra-pair discordance for global methylation was seen, older pairs were found to be twice as discordant as the younger twins [88]. This trend also held true for the disease-related loci, with older twins displaying discordance that was 1.4 to 2.7-fold greater, and a variation increasing proportionately with age (with very weak effects of changing cellular heterogeneity) [88]. Both global and locus-specific temporal increase in methylation discordance was confirmed by a longitudinal follow-up. The overall absolute global methylation differences between the twins were nonetheless small (1.1% in younger and 2.1% in older pairs). The results seem to confirm the earlier findings by Fraga *et al. *(2005) and suggest that discordance increases progressively with age [88]. The study points to the importance of unique individual environment behind the discordance, but explains the trend with both the influence of stochastic and environmental factors, acknowledging the difficulty in separating their effects from each other, and stressing that non-shared environmental exposures may also drive stochastic epigenetic discordance [88]. Table 1 sums up the main methylomic stability and discordance studies conducted in MZ twins.

**Table 1:** Studies of CpG methylation discordance in monozygotic (MZ) twins

Study	Type	Method	Tissue	MZ pairs, n	Conclusion
Fraga *et al. *[29]	Cross-sectional, age-stratified	High-performance capillary electrophoresis of total methyl-cytosine content	Peripheral lymphocytes; buccal epithelial cells; muscle biopsy; adipose tissue	40	Young MZ twins are nearly identical epigenetically; discordance progresses with age, mediated by a combination of external and/or internal factors·

Kaminsky *et al. *[85]	Cross-sectional	Human 12 K CpG island microarrays	White blood cells, buccal epithelial cells, rectal biopsy	57^1^	Methylation discordance in MZ twins confirmed;· monochorionic MZ twins significantly more discordant than dichorionic MZ twins^.^. Epigenetic drift suggested as the main cause of discordance^.^

Saffery *et al. *[86]	Cross-sectional, taken at birth	Bis-seq (*IGF2/H19*)	Cord blood, mononuclear cells, buccal epithelial cells, placental cells, umbilical vein cells, endothelial cells	56	CpG methylation discordance can arise in newborn twins by combination of environmental and/or stochastic factors acting *in utero *and varies depending on the type of the tissue^.^

Wong *et al. *[87]	Longitudinal, with single 5-year interval	High-throughput mass spectrometry (*DRD4, SERT, MAOA*)	Buccal cells, epithelial cells	46	CpG methylation discordance is present in early childhood and susceptibility to epigenetic change is highly locus-specific.^. ^Environmental influence is the main cause of discordance, with various loci having differential susceptibility to shared and non-shared exposures^.^

Talens *et al. *[88]	Cross-sectional and longitudinal with single 10-year interval	High-throughput mass spectrometry; global methylation and selected loci (*IGF2*, *LEP*, *CRH*, *ABCA1*, *INS*, *KCNQ1OT1*, *GNASAS)*	Whole blood	230	Global and locus-specific methylation increases gradually with age, owing to unique environmental and stochastic factors^.^

Abbreviations: MZ, monozygotic; Bis-seq, bisulfite sequencing

^1^Not all pairs were used for each estimate in the study

Together, these findings suggest that methylation patterns can be to a large extent genetically determined and heritable, yet do not remain stable over a person's lifetime. Variable degrees of epigenetic discordance can be seen in MZ twins, and it is evident that sample size, age, tissue type and CpG island selection can all significantly influence its estimates. There is substantial locus-to-locus and inter-individual variation in temporal methylation dynamics. To date, there is conflicting evidence on early epigenetic discordance in MZ twins, but age should be a crucial factor in all future studies of methylome changes. Chorionicity seems to be an important factor altering discordance and heritability estimates, therefore studies investigating methylome concordance in twins should also take this into account, although this information is often lacking. One potential problem that can affect findings in longitudinal studies is resampling from epigenetically different cellular subpopulations [87]. The environmental influence on the epigenome is relevant, and global epigenome studies in human MZ twins alone cannot resolve the sources of epigenetic discordance. Because the intrauterine environment, post-natal shared and non-shared environmental factors, and sequence polymorphisms acting in *cis *and *trans *can all be partly responsible for the methylation discordance, evaluating the significance of the intrinsic, stochastic epigenetic drift poses a methodological obstacle that is difficult to surmount [4,29,73,86,87,89,90]. Estimating the exact proportion of stochastically determined differences will require a deeper knowledge of the ways in which the non-shared environment shapes the methylome and the specific mechanisms responsible for the drift [29]. These may be difficult to investigate in humans.

## Methylation studies and human disease

A number of studies have investigated methylation differences in MZ twins in relation to disease or different phenotypic conditions (Table 2). Initially, studies utilized bisulfite conversion combined with sequencing of pre-selected candidate loci. In some of the very first twin methylation studies Petronis *et al. *found differences in the CpG methylation of a regulatory sequence of the dopamine D2 receptor, and this was greater in a schizophrenia-discordant pair than in a concordant one [91]. Another early study using bisulfite sequencing found methylation discordance at two regions of the *COMT *gene promoter in a sample of six MZ twin pairs, all discordant for birth weight [92]. Oates *et al. *(2006) used bisulfite sequencing of the *AXIN1 *promoter in a MZ pair discordant for caudal duplication anomaly [93]. More recently, a survey of CpG methylation of six chosen tumor-suppressor genes (*ATM*, *BRCA1*, *BRCA2*, *MLH1*, *RAD51C *and *TP53*) in a single MZ twin pair discordant for childhood leukemia and secondary thyroid carcinoma, identified increased *BRCA2 *methylation [82]. The proband had a significantly more methylated promoter than the healthy co-twin. The main limitations of these studies are the small sample sizes and narrow scope, limiting the number of potential associations to be found.

**Table 2:** Methylation studies in monozygotic (MZ) twins discordant for personality and disease

Study	Condition	Method	Tissue	MZ pairs, n	Results
Weksberg *et al. *[83]	Beckwith-Wiedemann syndrome	Southern blotting with DMR probes (*H19, KvDMR1, SNRP*)	Lymphocytes and fibroblasts	10	Loss of methylation at *KvDMR1 *in all probands

Petronis *et al. *[91]	Schizophrenia	Bis-seq (*DRD2*)	Lymphocytes	1	Discordance confirmed

Mill *et al. *[92]	Attention deficit hyperactivity disorder	Bis-seq (*COMT*)	Buccal epithelial cells	12	0.1 to 52.3% discordance

Oates *et al. *[93]	Caudal duplication	Bis-seq (*AXIN1*)	PBMC	1	Discordance confirmed

Kuratomi *et al. *[95]	Bipolar disorder	MS-RDA	Lymphoblastoid cell lines	1	4 DMRs, 1 candidate gene

Kaminsky *et al. *[94]	Risk-taking behavior	MEDIP-chip	PBMC	1	38 DMRs, 1 candidate gene

Mastroeni *et al. *[32]	Alzheimer disease	Immunohistochemistry	Temporal neocortex	1	Discordance confirmed

Javierre *et al. *[96]	Systemic lupus erythematosus	MEDIP-chip	White blood cells	5	49 DMRs, 8 candidate genes

Wong *et al. *[87]	ADHD, depression, antisocial behavior	Quantitative high-throughput mass spectrometry (*DRD4, SERT, MAOA*)	Buccal epithelial cells	46	Discordance confirmed

Baranzini *et al. *[72]	Multiple sclerosis	RRBS	CD4+ lymphocytes	3	2-178 DMRs, no candidate

Hu *et al. *[98]	Autism	MEDIP-chip	Lymphoblastoid cell lines	3	73 DMRs, 2 candidate genes

Tierling *et al. *[118]	Beckwith-Wiedemann syndrome	Bis-seq (11 DMR)	Peripheral blood cells, buccal epithelial cells, skin fibroblasts, saliva	1	Hypomethylation at *KvDMR1*

Harder *et al. *[119]	Optic glioma	Bis-seq (NF1)	Leukocytes	8	Discordance confirmed

Souren *et al. *[120]	BMI	Bis-seq	Saliva	8	Small discordance identified, not correlated with BMI discordance

Rakyan *et al. *[90]	Type 1 diabetes	Illumina Array	CD14+ cells	15 (+9 healthy control)	132 methylation variable positions

Dempster *et al. *[99]	Schizophrenia/Bipolar disorder	Illumina Array	Whole blood	22	Disease-associated DMRs, including *ST6GALNAC1 *as the top candidate

Galetzka *et al. *[82]	Childhood leukemia/secondary thyroid carcinoma	Bis-seq (6 tumor suppressors)	Skin fibroblasts	1	Increased *BRCA1 *methylation

Gervin *et al. *[100]	Psoriasis	Illumina Array	CD4+ and CD8+ cells	27	No significant methylation difference. Significant correlation between some DMRs and psoriasis-associated gene expression differences

Abbreviations: Bis-seq, bisulfite sequencing; BMI, body mass index; DMR, differentially methylated region; MS-RDA, Methylation-sensitive representational difference analysis; PBMC, peripheral blood mononuclear cell; RRBS, reduced representation bisulfite sequencing; MeDIP-chip, Methylated DNA immunoprecipitation - chip

With the advance of microarray and next-generation sequencing technology, it became possible to study methylation changes on a genome-wide scale. In a study of discordant risk-taking attitudes in a single MZ twin pair, Kaminsky *et al *(2007) looked at CpG methylation of about 12,192 CpG loci and found differences in methylation of the *DLX1 *gene, implicated in stress-response [94]. A methylation-sensitive-representational difference analysis study on a MZ pair discordant for bipolar disorder by Kuratomi *et al. *yielded four DMRs and one candidate gene, also confirmed to be differentially expressed [95]. Using an Illumina GoldenGate array, Javierre *et al *(2009) looked at methylation of 1505 CpG sites in 807 gene promoters across five MZ twins discordant for systemic lupus erythematosus (SLE), five twins discordant for rheumatoid arthritis (RA) and five twins discordant for dermatomyositis (DM), and discovered significant differences at 49 loci between the SLE-affected twins and their healthy co-twins, which were not seen in the RA and DM discordant twins [96]. The SLE cases had lower methylation levels and higher expression in several genes with immune functions. In a more recent analysis, Baranzini *et al. *used reduced representation bisulfite sequencing to investigate differences in methylation state of approximately 2 million CpG dinucleotides in three MZ twin pairs discordant for multiple sclerosis) [72]. The authors used high thresholds for methylation differences, which reduced the number of differentially methylated loci to 2, 10 and 176 between the different twin pairs. The differences were inconsistent between the three twin pairs, leading the authors to conclude that methylation differences could not explain twin discordance. The study's small sample size and its heterogenous character (twins of Ashkenazi Jewish African American and European descent) constituted perhaps the greatest limitation reducing the power to detect significant methylation differences [97]. A recent analysis of methylation in three pairs of MZ twins discordant for autism using an 8.1 K CpG microarray yielded 73 differently methylated CpG islands and two candidate genes [98]. The first genome-wide study using an Illumina 27 k array (covering ~27,000 CpG sites) of MZ twin methylomes in schizophrenia and bipolar disorder in a cohort of 22 discordant pairs revealed a number of disease-associated DMRs, including *GGN*, *SLC117A*, *SMUG1*, *SOX1 *and *TCF7L2*, which had been implicated in a previous study [99]. Methylome profiling with the Illumina 27 k array in a MZ twin cohort discordant for psoriasis failed to identify any significant DMRs, but did show that methylation correlated with the levels of expression at some disease-associated loci, including *IL13, ALOX5AP, PTHLH and TNFSF11 *[100]. A slightly different approach was adopted by Mastreoni *et al.*, who used immunohistochemistry to investigate whole-tissue methylation [32]. Different methylation levels in temporal neocortex neuronal nuclei were found in two MZ twins discordant for Alzheimer disease, with hypomethylation in the affected twin [32].

Although genome-wide studies have enabled discovery of more DMRs, such studies are still in their infancy, and face a number of issues [101]. To date, most studies have investigated methylation in small samples of one to a dozen twin pairs; use of larger discordant MZ twin cohorts will increase the power to detect potentially causal DMRs. However, increasing the size of the twin sample might be challenging for rare diseases and study designs involving longitudinal sampling [101]. Improvements to study designs in the future will probably require sampling from multiple tissues, particularly those that might be relevant to disease, because variation in the epigenome varies significantly across different cell types, and tissue-specific epimutations may play more important roles than systemic epimutations. However, some tissues are not easily accessible, and sampling from different tissues might involve biopsy and post-mortem material [101]. This is an important limitation, and some of the recent studies assayed methylation differences in tissues that were not directly relevant to the disease investigated. Currently, the use of several technologies and platforms makes crosscomparisons difficult [101,102]. Comparisons between MZ and DZ and between MC and DC twins should provide insights into the role of genetics and intrauterine environment in shaping epigenetic variation.

## Disease studies and causality

The associations yielded by various methylation studies emphasize the need to develop methods that establish causality [101,102]. Traditionally, in genetic studies, this was achieved by demonstrating perfect co-segregation of putative causal alleles with affected individuals in families, as well as alterations to the expression or structure of the protein encoded by the allele [103,104]. In non-mendelian complex diseases with a significant environmental component and no clear-cut disease segregation, causality is mainly investigated through case-control association studies by sorting candidate genes using *P*-value thresholds that minimize false-positive errors, and optimally by replicating the results in independent cohorts [105-107]. Of course, owing to linkage disequilibrium, population stratification, type I and type II errors, or mere chance, association does not equate to causality until proven by functional work [106,108]. Proving causality is, just like the definition, ultimately always context-dependent, and there is no uniform agreement on what constitutes adequate evidence; however, most authors are clear that some physical, biological link ought to be established [107,109-114]. Epigenetic alterations at promoter sites should affect transcriptional activity [94,98]. However, the problem is that epigenetic differences could in fact be side-effects of disease or treatment. Studies investigating epigenetic changes are potentially prone to false conclusions as a result of reverse causation or confounding [115]. Because the nature of the epigenome is dynamic and most epimutations arise throughout a person's lifetime, the key to addressing causality might be in their timing [97]. A longitudinal approach assaying for epigenetic discordance at birth or in early infancy and resampling at regular intervals should produce a timeline for methylation changes, and help to sort the potentially causal alterations from the secondary, side-effect ones. Epigenetic differences identified in twins could be further investigated in longitudinal cohorts as part of a two-stage study design [115]. Ultimately, studies of disease-associated epigenetic changes should be followed up with the aim of establishing a link with biological function [115]. To date, only one longitudinal epigenome-wide study has been conducted, investigating single CpG methylation differences in a panel of MZ twins discordant for type 1 diabetes. [116] In this study, 132 methylation variable positions associated with disease status were discovered, some of which were replicated in an independent group of nine singletons affected with type 1 diabetes affected singletons prior to disease diagnosis. [116]

## Conclusion

The plausible assumption made by Galton[117] that twin discordance can be explained by differential environmental exposures after birth is no longer tenable. Genetics, the *in utero *environment, stochastity, and epigenetics can all potentially play a role in determining phenotypic discordance. The field of epigenetics is in its infancy. There is very strong evidence for the direct role and relevance of epigenetics in shaping human phenotypic variability. The role of the epigenome can be both as a mediator of genetic and environmental effects or as an independent stochastic factor. Currently, the significance of primary epimutations in twin discordance is unknown. Furthermore, it is not fully clear as to what extent the epigenome is heritable and whether monozygotic twins are epigenetically identical at birth. Further studies are required to address these important questions. Ultimately, longitudinal studies with repeated sampling may be required to fully understand the nature of monozygotic twin discordance.

## List of Abbreviations

BMI: body mass index; CNV: copy number variant; DA: diamniotic; DAE: differential allelic expression; DC: dichorionic; DM: dermatomyositis; DMR: differentially methylated region; DZ: dizygotic; UGR: intrauterine growth restriction; MA: monoamniotic; MC: monochorionic; MZ: monozygotic; RA: rheumatoid arthritis; RRBS: reduced representation bisulfite sequencing; SLE: systemic lupus erythematosus; TTTS: twin-to-twin transfusion syndrome.

## Competing interests

The authors declare that they have no competing interests.

## Authors' contributions

WC and SVR designed the review and concepts, SVR and GCE were study supervisors, WC drafted the manuscript, JMM provided critical review of the manuscript. All authors read and approved the final manuscript.

## Funding

This work was funded by the Wellcome Trust [090532/Z/09/Z].

## Pre-publication history

The pre-publication history for this paper can be accessed here:

http://www.biomedcentral.com/1741-7015/10/93/prepub
